# Metallation of Isatin (2,3-Indolinedione). X-Ray Structure and Solution Behavior of Bis(Isatinato)Mercury(II)

**DOI:** 10.1155/MBD.1995.81

**Published:** 1995

**Authors:** Angel Garcia-Raso, Juan J. Fiol, Elies Molins, Antonia M. Calafat, Patricia A. Marzilli, Luigi G. Marzilli

**Affiliations:** 1 Department of Chemistry Universitat de les Illes Balears Palma de Mallorca 07071 Spain; 2 Institut de Ciencia de Materials de Barcelona-CSIC Campus Universitari de Bellaterra Cerdanyola 08193 Spain; 3 Department of Chemistry Emory University Atlanta Georgia 30322 USA

## Abstract

The first X-ray structure of an isatin (2,3-indolinedione, isaH) metal complex, bis(isatinato)memury(II) (C_16_H_8_N_2_O_4_Hg) (1), was determined. (1) was obtained from the reaction of isaH with mercury(II) acetate in methanol. Analogously, treatment of sodium saccharinate and mercury(II) acetate in methanol yielded Hg(saccharinato)_2_^•^0.5CH_3_OH (3). (1) crystallizes in the monoclinic system, space group *P*2_1_/ *a* with *a* = 7.299(1) Å, *b* = 8.192(1) Å, *c* = 11.601(1) Å , β = 105.82(1)°, *V* = 667.4 Å^3^, *Z* = 2, D_calc_ = 2.452 g cm^−3^, MoKα radiation(λ = 0.71073 Å), μ = 115.5 cm^-1^, *F*(000) = 460, 21(1) °C. The structure was refined on the basis of 2023 observed reflections to *R*= 0.044. The two deprotonated, non coplanar isa ligands are trans to each other in a head to tail orientation and bound to the Hg through the nitrogen in a linear N-Hg-N arrangement. The Hg atom is at the center of symmetry of the complex and displaced by 0.62 Å from the two planes of the isa ligands (τ Hg-N1-C2-O2= -16°). The Hg-N bond length is 2.015 Å. Noπ-aryl-memury(ll)-π-aryl stacking interaction was observed either in the solid state or in the solution state. The IR, electronic, and ^1^H and ^13^CNMR spectral data of (1) and (3) suggest binding of the memury to the heterocyclic nitrogen, in agreement with the crystal structure determination of (1).


### METALLATION OF ISATIN (2,3-INDOLINEDIONE). X-RAY STRUCTURE AND SOLUTION BEHAVIOR OF BIS(ISATINATO)MERCURY(II)

Angel Garcia-Raso,[1] Juan J. Fiol,[1] Elies Molins,[2] Antonia M. Calafat,[3]
Patricia A. Marzilli,[3] and Luigi G. Marzilli[3]

[1] Department of Chemistry, Universitat de les Illes Balears, 07071 Palma de Mallorca, Spain
[2] Institut de Ciencia de Materials de Barcelona-CSIC,
Campus Universitari de Bellaterra, Cerdanyola 08193, Spain
[3] Department of Chemistry, Emory University, Atlanta, Georgia 30322, USA

**ABSTRACT.** The first X-ray structure of an isatin (2,3-indolinedione, isaH) metal complex, bis(isatinato)mercury(II) ($C_{16}H_8N_2O_4Hg$) (**1**), was determined. (**1**) was obtained from the reaction of isaH with mercury(II) acetate in methanol. Analogously, treatment of sodium saccharinate and mercury(II) acetate in methanol yielded Hg(saccharinato)$_2$•0.5$CH_3OH$ (**3**). (**1**) crystallizes in the monoclinic system, space group $P2_1/a$ with $a$ = 7.299(1) Å, $b$ = 8.192(1) Å, $c$ = 11.601(1) Å, $\beta$ = 105.82(1)°, $V$ = 667.4 Å$^3$, $Z$ = 2, $D_{calc}$ = 2.452 g cm$^{-3}$, MoK$\alpha$ radiation ($\lambda$ = 0.71073 Å), $\mu$ = 115.5 cm$^{-1}$, $F$(000) = 460, 21(1) °C. The structure was refined on the basis of 2023 observed reflections to $R$ = 0.044. The two deprotonated, non coplanar isa ligands are trans to each other in a head to tail orientation and bound to the Hg through the nitrogen in a linear N-Hg-N arrangement. The Hg atom is at the center of symmetry of the complex and displaced by 0.62 Å from the two planes of the isa ligands ($\tau$ Hg-N1-C2-O2 = -16°). The Hg-N bond length is 2.015 Å. No $\pi$-aryl-mercury(II)-$\pi$-aryl stacking interaction was observed either in the solid state or in the solution state. The IR, electronic, and $^1$H and $^{13}$C NMR spectral data of (**1**) and (**3**) suggest binding of the mercury to the heterocyclic nitrogen, in agreement with the crystal structure determination of (**1**).

### INTRODUCTION

Interactions of heavy metal ions with biomolecules are of interest because of their potential toxic effects. However, the mechanism of action and the biological speciation of toxic metal species are not well defined, although interaction with DNA may be involved. The reversible binding of mercury to DNA has generated some interest, but is still poorly understood at the molecular level. Katz proposed a chain slippage mechanism in which Hg(II) crosslinked thymine (thyH) nucleobases in opposite strands of the [polyd(AT)]$_2$ duplex by forming N3-Hg-N3 bonds, thus disrupting the Watson-Crick hydrogen bonds.[1] It is widely accepted that mercury binds to the DNA bases, and preferentially to N3 of thymidine as compared to N1 of guanosine.[2,3] NMR studies of the interaction between Hg(II) and synthetic oligonucleotides indicate that the thy N3H $^1$H signals disappear upon Hg(II) complexation,[4,5] supporting Katz's proposal. The propensity of Hg(II) to bind to highly basic, mononegative sp$^2$ nitrogens could also be manifest in pharmaceuticals or food additives.

Isatin (2,3-indolinedione, isaH) and some of its derivatives, e.g. thiosemicarbazones, possess antiviral activity which is often enhanced upon complexation with metal ions.[6-8] Although some transition metal complexes with Schiff bases derived from isatin have been studied,[9,10] to our knowledge no metal-isatin crystal structure has been reported. The use of saccharin (1,2-benzisothiazol-3(2H)-one 1,1-dioxide, saccH) as an artificial sweetener is widespread. At high dose levels induces bladder cancer in rats. Some of the saccharin halogeno derivatives are believed to have fungicidal activity, N-acylsaccharins are elastase inhibitors, and the acetic acid derivative is a potent sedative, hypnotic and anticonvulsant.[11] These two species (Scheme 1) have NH groups that can readily be deprotonated and bind to Hg(II). They are thus relevant to understanding Hg(II) binding to DNA. Therefore, the study of the coordination properties of isaH and saccH is of considerable interest. We report the preparation, spectroscopic characterization and the first crystallographic study of an isatin complex, Hg(isa)$_2$, (**1**).

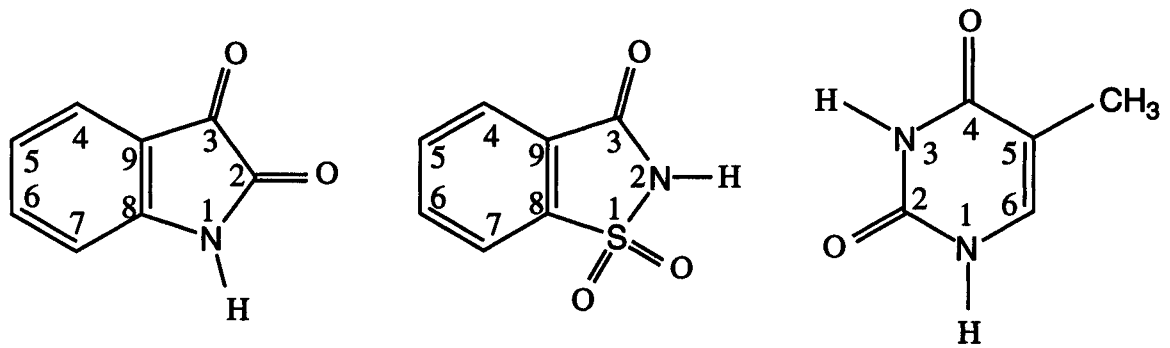

isaH saccH thyH

**Scheme 1**

### MATERIALS AND METHODS

### *Analyses and Physical Measurements*

Isatin and mercury(II) acetate were obtained from Merck and used without further purification; sodium saccharinate was from Aldrich. Neutral saccharin was obtained from sodium saccharinate by acidification with HCl to pH 2. The precipitate was removed by filtration and washed with cold $H_2O$. Elemental analyses were carried out in a Carlo Erba model 1106 microanalyzer (Centro de Investigaci6n y Desarrollo-CSIC, Barcelona, Spain). Thermogravimetric studies were carried out with a PE TGA-2 thermobalance with oxygen atmosphere (heating rate 5°C/min). Infrared spectra were recorded in the solid state (KBr pellets) on a PE 683 spectrometer with an infrared data station PE 1600, and the electronic spectra were recorded on a PE 552 spectrophotometer. The 1D NMR spectral measurements of isaH and (1) were made using Gemini 200 and Bruker AMX-300 spectrometers. The 1D $^1H$ and $^{13}C$ NMR spectra of sacc, saccH and (3) were carried out on an Omega GN-600 spectrometer. Proton and carbon chemical shifts in DMSO-$d_6$ were referenced to internal $Me_4Si$. The 2D NMR experiments were performed on an Omega GN-600 spectrometer at 25 °C without sample spinning. $^1H$-Detected Heteronuclear Multiple Quantum Coherence Spectroscopy (HMQC)[12,13]: The one-bond $^1H$-$^{13}C$ shift correlation spectra resulted from a 256 x 2048 data matrix size with 64, 176, 100, and 100 scans (preceded by 4 dummy scans) per $t_1$ value (for $Hg(isa)_2$, isaH, $Hg(sacc)_2$, and Na(sacc), respectively). Predelay was 1.1 s, except for isaH and $Hg(sacc)_2$ (1.0 s), and 66W (71 dB) of $^{13}C$ rf power and a 31-µs 90° $^{13}C$ pulse width were used. A sine bell squared filter was used prior to Fourier transformation in the $t_2$ and $t_1$ dimensions. $^1H$-Detected Multiple-Bond Heteronuclear Multiple Quantum Coherence Spectroscopy (HMBC)[14]: The multiple-bond $^1H$-$^{13}C$ shift correlation spectra resulted from a 256 x 2048 data matrix size with 160, 160, 256, 208, and 176 scans (preceded by 4 dummy scans) per $t_1$ value (for $Hg(isa)_2$, $Hg(sacc)_2$, isaH, saccH, and Na(sacc), respectively). Predelay was 1.1 s, except for isaH and $Hg(sacc)_2$ (1.0 s), and 66W (71 dB) of $^{13}C$ rf power and a 31-µs 90° $^{13}C$ pulse width were used. The delay between the first 90° $^1H$ pulse and the first 90° $^{13}C$ pulse was 3.3 ms. The delay between the first and the second 90° $^{13}C$ pulses was 53.3 ms. A sine bell squared filter was used prior to Fourier transformation in both $t_1$ and $t_2$ dimensions. The 2D spectra were processed by using the Felix program (Hare Research, Inc.).

### *Preparative Method*

The mixture of a methanol solution of isaH and a methanol solution of mercury(II) acetate (2:1) afforded an orange precipitate, which was filtered and washed with methanol to give (1). Crystals suitable for X-ray diffraction were obtained from attempts to crystallize mixed-metal Hg/lanthanide complexes in DMSO/methanol (1/20) in the presence of a 0.5 mole ratio of praseodymium nitrate.[15] Anal. Calcd for $C_{16}H_8N_2O_4Hg$: C, 38.98; H, 1.62; N, 5.68. Found: C,

38.98; H, 1.62; N, 5.67. IR($cm^{-1}$): 314 w, 479 m, 490 w, 690 m, 761 m, 821 w, 868 w, 919 m, 972 m, 1092 w, 1185 w, 1217 m, 1278 m, 1313 m, 1334 m, 1458 m, 1596 s, 1675 s, 1730 s. (s = strong, m = medium, w = weak). $\Lambda_M$ ($\Omega^{-1}$ $cm^2$ $mol^{-1}$) = 2.4 for $10^{-3}$ M solution in DMSO, 20 °C .

An analogous complex, the dihydrate derivative $Hg(isa)_2{\bullet}2H_2O$ (**2**), was obtained as red crystals after mixing methanol solutions of (**1**) and $Co(NO_3)_2{\bullet}6H_2O$ in a 2:1 molar ratio. Unfortunately, the crystals were not suitable for X-ray analysis. Anal. Calcd for $C_{16}H_{12}N_2O_6Hg$: C, 36.32; H, 2.27; N, 5.30. Found: C, 36.39; H, 1.97; N, 5.31. IR ($cm^{-1}$): 295w, 315w, 476s, 552m, 647m, 668m, 693m, 758s, 815w, 918m, 973m, 1094m, 1190m, 1216s, 1302s, 1333m, 1462s, 1603s, 1683s, 1727s, 1750s.

$Hg(sacc)_2{\bullet}0.5\ CH_3OH$ (**3**) was prepared by mixing a methanol solution of sodium saccharinate and a methanol solution of mercury(II) acetate (2:1). A white precipitate that appeared immediately was collected and washed with methanol. A crystalline material was obtained from ethanol and DMSO/methanol (1/20) in the presence of a 0.5 mole ratio of praseodymium nitrate.

**TABLE I.** Crystal Data and Structure Solution Parameters for (**1**)

| | |
|---|---|
| complex | $Hg(isa)_2$ |
| formula | $C_{16}H_8N_2O_4Hg$ |
| fw | 492.83 |
| crystal system | monoclinic |
| space group | $P2_1/a$ |
| *a* (Å) | 7.299(1) |
| *b* (Å) | 8.192(1) |
| *c* (Å) | 11.601(1) |
| ß (deg) | 105.82(1) |
| *V* ($Å^3$) | 667.4 |
| *Z* | 2 |
| *F*(000) | 460 |
| Dc($g/cm^3$) | 2.45 |
| crystal color, habit | orange, prismatic |
| crystal dimens. (mm) | 0.34 x 0.27 x 0.04 |
| μ(MoKα) ($cm^{-1}$) | 115.5 |
| diffractometer | Enraf-Nonius CAD4 |
| radiation | MoKα (λ = 0.71073 Å) |
| corrections | Lorentz-polarization |
| | absorp. by Gaussian method |
| | (max/min transmission 0.63,0.12) |
| | Reflection average (agreement on I = 1.5%) |
| scan type | ω-2θ |
| $2\theta_{max}$ | 60.9° |
| range of *hkl* | $-10<h<0$, $0<k<11$, $-15<l<16$ |
| no. of reflns measured | 2304 total, 2158 unique |
| no. of reflns included | 2023 with $Fo>3.0\sigma(Fo)$ |
| solution | Patterson methods and weighted Fourier synthesis |
| hydrogen atoms | located and refined isotropically |
| parameters refined | 122 |
| residuals: *R*; $R_w$ | 0.044; 0.043 |
| esd of obs. of unit weight | 1.74 |
| convergence, largest shift | 0.30 |
| minimization function | $\Sigma w(\lvert F_o\rvert - \lvert F_c\rvert)^2$ |
| least-squares weights | $4F_o^2/\sigma(F_o^2)$ |
| instrument instability factor | 0.040 |
| high peak in final diff. map | 1.6(4) $eÅ^{-3}$ |
| low peak in final diff. map | -2.4(4) $eÅ^{-3}$ |

Anal. Calcd for $C_{14.5}H_{10}N_2O_{6.5}S_2Hg$: C, 29.97; H, 1.72; N, 4.82. Found: C, 30.06; H, 1.65; N, 4.72. IR ($cm^{-1}$): 352w, 402w, 462w, 533m, 545m, 611m, 637w, 680m, 709w, 752m, 774m, 794m, 880w, 955m, 965m, 1053m, 1119m, 1129m, 1154s, 1168s, 1254s, 1264s, 1289m, 1335m, 1350w, 1460w, 1584s, 1627s, 1659m. $\Lambda_M$ ($\Omega^{-1}$ $cm^2$ $mol^{-1}$) = 5.6 for $10^{-3}$ M solution in DMSO, 20 °C .

(**1**) was thermally stable up to 230 °C. From 230-330 °C, it exhibited a weight loss corresponding to the loss of the two isa moieties per formula unit (Mass loss: Calcd, 59.27%; Found, 58.33%). Further decomposition occurred between 330-530 °C, leaving no residue.

The thermal decomposition of (**2**) began with a dehydration process at ~120°C, corresponding to the elimination of two water molecules per formula unit (Mass loss: Calcd, 6.81%; Found, 6.25%). The pyrolytic decomposition of the dehydrated complex occurred in two main steps in a manner similar to that of (**1**). Again the volatilization of the sample was total.

(**3**) was thermally stable up to 170 °C. Between this temperature and 200 °C, it exhibited a weight loss corresponding to the loss of the 0.5 molecule of $CH_3OH$ (Mass loss: Calcd, 2.8%; Found, 2.8%). At ~390 °C, one sacc moiety was lost (Mass loss: Calcd, 32.4%; Found, 31.8%).

*X-Ray Data Collection and Structure Determination*

Table I lists the crystallographic data and details of the X-ray structure analysis for (**1**). Final positional parameters are listed in Table II.

**TABLE II.** Positional Parameters and Their Estimated Standard Deviations for (**1**)

| atom | x | y | z | $B_{eq}$ ($Å^2$) |
|---|---|---|---|---|
| Hg | 0 | 0.5 | 0 | 1.809(5) |
| O2 | 0.2777(8) | 0.8012(6) | 0.1178(5) | 3.2(1) |
| O3 | 0.5810(8) | 0.7443(7) | 0.3437(5) | 3.3(1) |
| N1 | 0.2496(8) | 0.5201(7) | 0.1273(5) | 2.08(9) |
| C2 | 0.324(1) | 0.6702(8) | 0.1663(6) | 2.3(1) |
| C3 | 0.4799(9) | 0.6399(8) | 0.2868(6) | 2.1(1) |
| C4 | 0.5746(9) | 0.3642(9) | 0.4012(6) | 2.5(1) |
| C5 | 0.543(1) | 0.1961(9) | 0.3905(7) | 2.9(1) |
| C6 | 0.416(1) | 0.1311(8) | 0.2864(6) | 2.5(1) |
| C7 | 0.3141(9) | 0.2310(8) | 0.1957(6) | 2.0(1) |
| C8 | 0.3415(8) | 0.3982(8) | 0.2089(6) | 2.0(1) |
| C9 | 0.4721(9) | 0.4640(8) | 0.3084(5) | 2.0(1) |
| H4 | 0.66(1) | 0.42(1) | 0.485(8) | 3(2)* |
| H5 | 0.61(1) | 0.12(1) | 0.455(8) | 4(2)* |
| H6 | 0.42(1) | 0.03(1) | 0.281(9) | 6(3)* |
| H7 | 0.24(1) | 0.18(1) | 0.125(7) | 2(2)* |

Starred atoms were refined isotropically. Anisotropically refined atoms are given in the form of the isotropic equivalent displacement parameter defined as:

$$B_{eq}=(8\pi^2/3)\Sigma_i\Sigma_j\ U_{ij}\ a_i^*\ a_j^*\ \vec{a_i}\bullet\vec{a_j}$$

A flat orange crystal with well-defined faces was selected for the X-ray diffraction experiment and mounted on a glass fiber. Cell parameters were obtained from 22 randomly searched high-order reflections. Once the crystal was oriented, the faces were indexed and accurately measured. Distances among parallel faces were 0.30 [110], 0.27 [110], 0.35 [100] and 0.04 mm [001]. Data were collected at 294(1) K. Three reflections were measured each hour without significant time decay. In order to account for the high absorption effects, some reflections were measured at each 10° of Ψ angle. The estimated maximum absorption was 91%. Lorentz and polarization corrections were applied. The best set of non-unique data was averaged ($R_{int}$ = 0.015), resulting in 2023

unique reflections. The Hg atom was located from a Patterson map and the remaining atoms, including hydrogens, appear in successive Fourier syntheses. In addition, several refinement processes were carried out using different sets of data corresponding to different absorption corrections.[16] The quality of the correction was estimated from the *R* value at convergence when all atoms were allowed to vibrate isotropically and, especially, by the height of the residual peak close to the Hg atom in a subsequent difference Fourier synthesis.

Empirical absorption corrections by 'psi scans', by 'Fourier series' or both applied consecutively gave good *R* values (about 0.04) but difference Fourier peaks between 8-17 $eÅ^{-3}$. Gaussian absorption correction was applied for different values of the crystal thickness δ (distance among [001] and [00 $\bar{1}$] faces). For δ values of 40 and 41 μm, the *R* value was 0.044 and the residual peak 1.7 $eÅ^{-3}$, whereas for δ values of 39 and 42 μm, *R* and the residual peak were 0.18 and 10.6 $eÅ^{-3}$, respectively. As expected, a Gaussian correction gave the best results, but only when crystal dimensions were evaluated accurately. Refinement with anisotropic thermal parameters for non-H atoms and isotropic for H-atoms converged at $R = 0.044$ ($R_w = 0.043$, $w = 4F_o^2/\sigma(F_o^2)$) using 1326 reflections with $F_o > 3\sigma(F_o)$.[17]

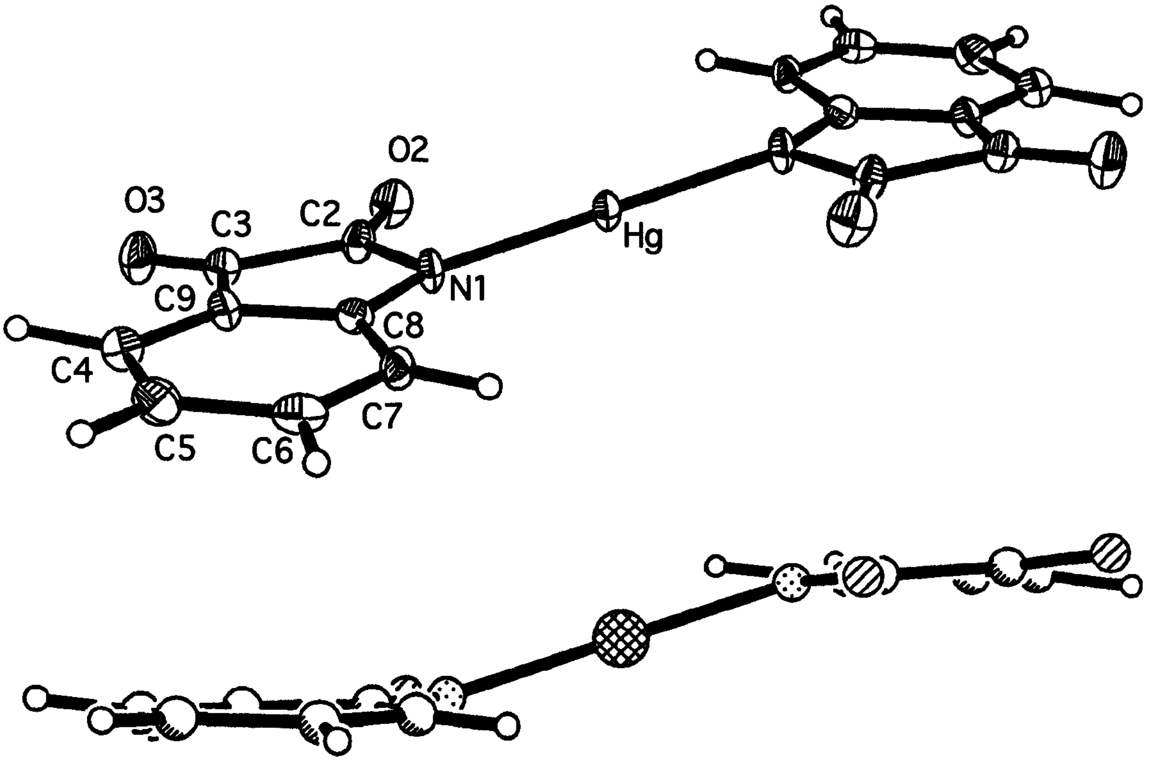

**FIGURE 1.** Molecular structure of $Hg(isa)_2$, showing the atom numbering scheme.

### RESULTS AND DISCUSSION

Reaction of isaH and saccH with mercury(II) acetate in methanol yielded the compounds $Hg(isa)_2$ (**1**) and $Hg(sacc)_2 \cdot 0.5CH_3OH$ (**3**), respectively. The molecular structure of (**1**) and the atom numbering scheme are shown in Figure 1. The two deprotonated, non coplanar isatin ligands are trans to each other and bound to the mercury in a head to tail orientation via the heterocyclic nitrogen in a linear N-Hg-N arrangement. The Hg-N bond distance (2.015(5) Å) is similar to previously reported distances in other Hg-N(heterocyclic) systems.[15,18-20] The Hg atom is at the center of symmetry of the complex and displaced by 0.62 Å from the two planes of the isa ligands

(torsion angle τ Hg-N1-C2-O2 = -16°). In $Hg(1\text{-methy})_2$, (1-methyH = 1-methylthymine)[20] the Hg atom is also at the center of symmetry of the complex but with the Hg only 0.3 Å above the thy ring. The Hg-N1-C2 and C2-N1-C8 angles of (1) contract to 120.8(4) and 109.9(5)°, respectively, and the Hg-N1-C8 angle enlarges to 127.0(4)° compared to the corresponding angles in the free isaH moiety.[21-23] These facts could be related to some distortion of the geometry at N1 upon Hg complexation, as described previously for a 4-methyl-2-(1H)-quinolone mercury (II) complex.[15] In contrast, no noticeable distortion at N3 was found in $Hg(1\text{-methy})_2$.[20] The very long C2-C3 bond (1.564(9) Å) of (1), also observed in the structure of the free ligand, has been related to nonbonded lone pair-lone pair repulsions in cis-diketones.[21] Selected bond distances and bond angles are given in Table III.

**TABLE III.** Selected Bond Lengths and Angles for Compound (1)[a]

Bond Lengths (Å)

| | | | | | |
|---|---|---|---|---|---|
| Hg | N1 | 2.015(5) | C4 | H4 | 1.11(9) |
| O2 | C2 | 1.215(8) | C5 | C6 | 1.41(1) |
| O3 | C3 | 1.203(8) | C5 | H5 | 1.0(1) |
| N1 | C2 | 1.370(8) | C6 | C7 | 1.38(1) |
| N1 | C8 | 1.414(8) | C6 | H6 | 0.8(1) |
| C2 | C3 | 1.564(9) | C7 | C8 | 1.386(8) |
| C3 | C9 | 1.467(9) | C7 | H7 | 0.94(7) |
| C4 | C5 | 1.40(1) | C8 | C9 | 1.390(8) |
| C4 | C9 | 1.396(9) | | | |

Bond Angles (deg)

| | | | | | | | |
|---|---|---|---|---|---|---|---|
| Hg | N1 | C2 | 120.8(4) | C6 | C5 | H5 | 120(6) |
| Hg | N1 | C8 | 127.0(4) | C5 | C6 | C7 | 121.4(6) |
| C2 | N1 | C8 | 109.9(5) | C5 | C6 | H6 | 114(8) |
| O2 | C2 | N1 | 127.2(6) | C7 | C6 | H6 | 124(8) |
| O2 | C2 | C3 | 126.6(6) | C6 | C7 | C8 | 118.0(6) |
| N1 | C2 | C3 | 106.2(5) | C6 | C7 | H7 | 118(4) |
| O3 | C3 | C2 | 124.5(6) | C8 | C7 | H7 | 124(5) |
| O3 | C3 | C9 | 130.6(6) | N1 | C8 | C7 | 126.6(6) |
| C2 | C3 | C9 | 104.9(5) | N1 | C8 | C9 | 112.0(5) |
| C5 | C4 | C9 | 118.0(7) | C7 | C8 | C9 | 121.4(6) |
| C5 | C4 | H4 | 123(5) | C3 | C9 | C4 | 132.0(6) |
| C9 | C4 | H4 | 118(5) | C3 | C9 | C8 | 106.8(5) |
| C4 | C5 | C6 | 120.1(7) | C4 | C9 | C8 | 121.0(6) |
| C4 | C5 | H5 | 120(6) | N1 | Hg | N1 | 180 |

[a] Numbers in parentheses are estimated standard deviations in the least significant digits.

The $Hg(isa)_2$ units are linked by hydrogen bond interactions (O2...H6 = 2.44 Å). This hydrogen bond network seems to play an important role in both the formation of a polymeric chain (Figure 2) and the stabilization of the crystal structure. Roughly speaking, the Hg atom is in a distorted octahedral environment with two Hg-N bonds of 2.015 Å and with two intramolecular and two intermolecular long Hg-O(2) contact distances of 3.256 and 2.899 Å, respectively. A similar situation was found in $Hg(1\text{-methy})_2$, where the Hg-O contacts were 3.03-3.05 Å.[20] The Hg of one $Hg(isa)_2$ unit lies between two adjacent $Hg(isa)_2$ chains, so π-aryl-Hg-π-aryl stacking interactions are not established, in contrast to what occurs in $Hg(4\text{-methyl-2-(1H)-quinolone})_2$.[15]

The infrared spectra of (1) and (3) in the far-IR region to 200 $cm^{-1}$ were compared with those of the free ligands. Tentative assignments of some bands were made by analogy with similar systems.[7,24,25] The disappearance of the broad νNH band at 3180 $cm^{-1}$ for isaH and at 3100 $cm^{-1}$

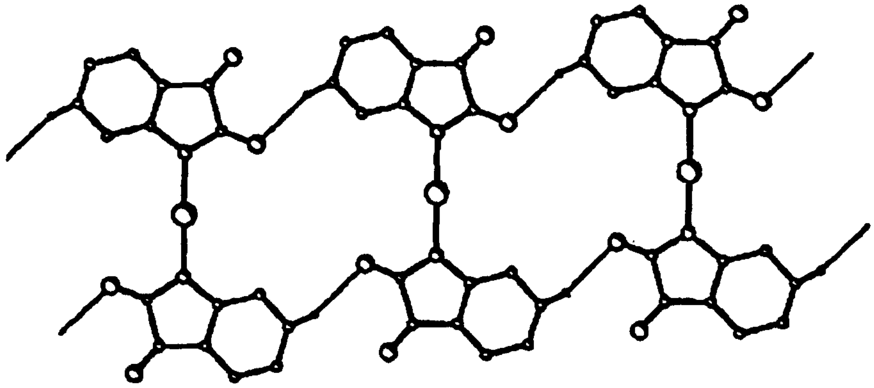

**FIGURE 2.** Drawing of the linked chains of $Hg(isa)_2$ units along *b*

for saccH suggests involvement of the heterocyclic nitrogen in the bonding in the respective complexes. Furthermore, the νC=O (1731 $cm^{-1}$) band of isaH split into two peaks at 1730 and 1675 $cm^{-1}$ and decreased in intensity. These changes would be consistent with the participation of the C(2)=O group in a hydrogen bond, as indicated by the crystallographic data. Most of the bands of isaH in the 1600-1300 $cm^{-1}$ region shift to lower frequencies in the spectrum of (**1**). For (**3**), the νC=O (1720 $cm^{-1}$) band of saccH split into two peaks at 1642 and 1627 $cm^{-1}$ and the 1336 and 1178 $cm^{-1}$ strong bands assignable to $\nu_a(SO_2)$ and $\nu_b(SO_2)$, respectively, shifted to lower frequencies. This is consistent with a considerable decrease in the bouble-bond character of the CO and $SO_2$ groups upon coordination.

The electronic spectrum of isaH in DMSO displays bands at 256 nm ($\varepsilon = 3.8x10^3$ $M^{-1}cm^{-1}$) and 297 nm ($\varepsilon = 3.63x10^3$) assignable to transitions of the benzene moiety. No changes in these bands were observed upon metal complex formation. In contrast, the characteristic charge-transfer band of the ligand at 418 nm ($\varepsilon = 1.07x10^3$) red-shifted to 445 nm ($\varepsilon = 1.38x10^3$) in the spectrum of (**1**). In the case of $Hg(sacc)_2$, the $\pi \rightarrow \pi^*$ transition band (272 nm, $\varepsilon = 1.42x10^3$) of the neutral saccharin in DMSO shifts to 256 nm ($\varepsilon = 2.4x10^4$) upon metal complex formation.

HMBC (Figure 3) and HMQC were used to assign the $^1H$ and $^{13}C$ NMR spectra of the ligands and the mercury complexes. The $^1H$ and $^{13}C$ assignments of isatin and $Hg(isa)_2$ in DMSO$d_6$ are shown in Table IV. The nonprotonated carbon resonances of (**1**) were recognized from the HMQC spectrum and assigned from the HMBC spectrum. The $^{13}C$ signal at 163.8 ppm, with no correlations in the HMBC spectrum, was assigned to C2. In order to complete $^1H$ and $^{13}C$ assignments, use was made of the fact that for aromatic systems three-bond $J_{CH}$ couplings are bigger than two bond couplings.[26] The doublet at 7.47 ppm with connectivities in the HMBC spectrum to the $^{13}C$ signals at 138.4, 158.3 and 186.3 ppm was assigned to H4. H4 is the only proton that can have three-bond correlations to the two nonprotonated carbon resonances at 158.3 and 186.3 ppm. The remaining doublet in the $^1H$ NMR spectrum at 7.38 ppm was then assigned to H7. C9 and C5 were assigned as the signals at 120.4 and 122.2 ppm from their HMBC correlations to H7. The triplet at 6.99 ppm was assigned to H5 from its HMQC correlation to C5, and the remaining triplet at 7.59 ppm was assigned to H6. C8 was assigned as the resonance at 158.3 ppm from its HMBC correlations to both H4 and H6. The remaining nonprotonated $^{13}C$ signal at 186.3 ppm was assigned to C3. Assignment of the $^1H$ and $^{13}C$ NMR spectra of isaH, saccH, Na(sacc)and $Hg(sacc)_2$ in DMSO-$d_6$ was done in an analogous way (Table IV).

Upon complexation of both isaH and saccH, the pattern of shifts, especially $^{13}C$ shifts can be readily categorized by the five-membered ring C's and the $(CH)_4$ group completing the six-membered ring. Significant downfield shifts were observed for the C2 and C8 resonances of isa. In addition, the isaH imino $^1H$ signal disappeared (Table IV). These changes are consistent with binding of mercury to isa through the heterocyclic nitrogen, as suggested by the X-ray structure. The large effects are all on the five-membered ring except for that on C7. The downfield shift for the C7 and H7 resonances of isa could be related to the proximity of the Hg to C7. In (**1**) the shortest

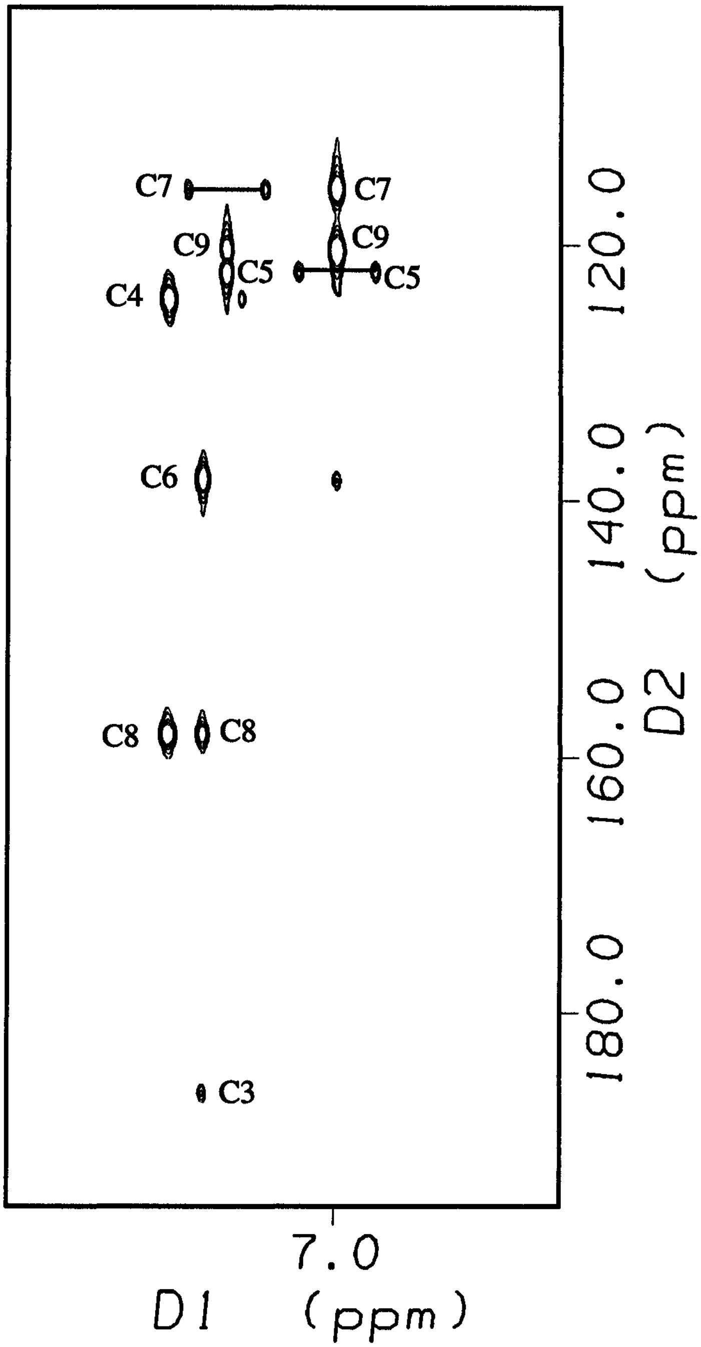

**FIGURE 3.** $^1H$-detected $^1H$-$^{13}C$ multiple-bond shift correlation (HMBC) spectrum of $Hg(isa)_2$ in DMSO-$d_6$. Incompletely suppressed "satellite" peaks from C5 and C7 are also present.

intramolecular nonbonded mercury contact to a CH is to C7H. No significant changes in $^1H$ and $^{13}C$ chemical shifts were observed for C4, C5 and C6 upon complexation, suggesting that in solution the aromatic rings are not involved in stacking interactions.

Protonation of saccharinate induced large upfield (~6-7.2 ppm) and significant downfield shifts (~2.2-4.7 ppm) for the five-membered ring and $(CH)_4$ $^{13}C$ resonances, respectively (Table IV). In addition, the $^1H$ resonances shifted downfield (~0.32-0.47 ppm) and a new signal in the imino proton region at 12.29 ppm appeared. The same NMR pattern, although with smaller shift

**TABLE IV.** $^1H$ and $^{13}C$ NMR Shift Assignments of isaH, $Hg(isa)_2$ (**1**), saccH, Na(sacc) and $Hg(sacc)_2$ (**3**) in DMSO-$d_6$[a]

| Assignment | $^1H$ (**1**)[b] | $^{13}C$ (**1**)[c] | $^1H$ isaH[b] | $^{13}C$ isaH[c] | $\Delta\delta(^{13}C)$[d] | $\Delta\delta(^1H)$[d] |
|---|---|---|---|---|---|---|
| N1H | | | 11.05 | | | |
| C2 | | 163.8 | | 159.3 | 4.5 | |
| C3 | | 186.3 | | 184.4 | 1.9 | |
| C4H | 7.47 | 124.3 | 7.51 | 124.7 | -0.4 | -0.04 |
| C5H | 6.99 | 122.2 | 7.07 | 122.7 | -0.5 | -0.08 |
| C6H | 7.59 | 138.4 | 7.59 | 138.3 | 0.1 | 0 |
| C7H | 7.38 | 115.7 | 6.92 | 112.2 | 3.5 | 0.46 |
| C8 | | 158.3 | | 150.7 | 7.6 | |
| C9 | | 120.4 | | 117.8 | 2.6 | |

| Assignment | $^1H$ (**3**)[b] | $^{13}C$ (**3**)[c] | $^1H$ saccH[b] | $^{13}C$ saccH[c] | $^1H$ sacc[b] | $^{13}C$ sacc[c] |
|---|---|---|---|---|---|---|
| N2H | | | 12.29 | | | |
| C3 | | 163.8 | | 160.7 | | 167.8 |
| C4H | 8.00 | 124.4 | 8.09 | 124.9 | 7.76 | 122.4 |
| C5H | 7.93 | 134.0 | 8.00 | 134.8 | 7.68 | 131.4 |
| C6H | 7.99 | 134.7 | 8.06 | 135.6 | 7.68 | 130.9 |
| C7H | 8.12 | 120.7 | 8.24 | 121.2 | 7.77 | 119.0 |
| C8 | | 142.3 | | 139.2 | | 145.2 |
| C9 | | 128.8 | | 127.5 | | 134.7 |

[a] $^1H$ and $^{13}C$ assignments were done by 2D NMR. [b]Relative to TMS. [c] $^{13}C$ shifts from the 1D NMR spectrum relative to TMS with DMSO-$d_6$ peak as reference (39.5 ppm). [d] $\Delta\delta = \delta_{(1)} - \delta_{(isa)}$

changes and without an imino $^1H$ resonance, were observed upon Hg interaction with sacc. All these facts are consistent with binding of the five-membered heterocyclic ring nitrogen to the Hg, in agreement with the crystal structure of $Hg(sacc)_2$.[27,28]

The shift pattern observed for $Hg(isa)_2$ compared to isaH and $Hg(sacc)_2$ compared to saccH both can probably best be viewed as the result of a less powerful electron deficient center ($Hg^{2+}$) vs. a more powerful electron deficient center ($H^+$).[29-31] Thus, the effects of $Hg^{2+}$ on the correctly ionized form of the free ligand, represented in this work by $sacc^-$, is less than that of the $H^+$. These effects are also evident in the binding of Hg(II) to thymidine, where the $Hg^{2+}$ center appears to cause a downfield shift of the C4 relative to thymidine.[2,3] Again, the best way to view the shifts is a smaller upfield shift in C4 of N3 deprotonated thymidine induced by $Hg^{2+}$ than by $H^+$.

In summary, we have established the structure of $Hg(isa)_2$ in solution and solid state and have demonstrated that, as found previously for DNA, NMR spectroscopy is useful in assessing speciation of Hg(II) with other classes of molecules such as pharmaceuticals and food additives.

### ACKNOWLEDGMENTS

We are grateful to DGICYT Ref.PB91-0806-C02-02 and the National Institutes of Health (Grant GM 29222 to L. G. M.) for financial support. We also wish to express our gratitude to Prof. V. Moreno, Universitat de Barcelona, Spain, for the 1D $^1H$ and $^{13}C$ NMR spectra of (**1**).

### REFERENCES

(1) Katz, S. *Biochim. Biophys. Acta* 1963, **68**, 240-253.
(2) Buncel, E.; Boone, C.; Joly, H.; Kumar, R.; Norris, A. R. *J. Inorg. Biochem.* 1985, **25**, 61-73.
(3) Buncel, E.; Boone, C.; Joly, H. *Inorg. Chim. Acta* 1986, **125**, 167-172.
(4) Frøystein, N. Å.; Sletten, E. *J. Am. Chem. Soc.* 1994, **116**, 3240-3250.

(5) Young, P. R.; Nandi, U. S.; Kallenbach, N. R. *Biochemistry* 1982, **21**, 62-66.
(6) Bauer, D. J.; Sadler, P. W. *Nature (London)* 1961, **190**, 1167.
(7) Hassaan, A. M. A. *Transition Metal Chemistry* 1990, **15**, 283.
(8) Patel, S. P.; Ray, A.; Patel, R. P. *Synthesis and Reactivity in Inorganic and Metal-Organic Chemistry* 1987, **17**, 419-430.
(9) Khulbe, R. C.; Singh, R. P.; Bhoon, Y. K. *Transition Metal Chemistry* 1983, **8**, 59.
(10) Garg, B. S.; Singh, R. P.; Garg, S. K. *Indian J. Chem.* 1991, **30A**, 979.
(11) Davis, M. *Advances in Heterocyclic Chemistry* 1985, **38**, 120.
(12) Muller, L. *J. Am. Chem. Soc.* 1979, **108**, 2093.
(13) Bax, A.; Subramanian, S. *J. Magn. Reson.* 1986, **67**, 565.
(14) Bax, A.; Summers, M. F. *J. Am. Chem. Soc.* 1986, **108**, 2093.
(15) Goodgame, D. M. L.; Hill, S. P. W.; Williams, D. J. *Polyhedron* 1992, **11**, 1507.
(16) Walker, N.; Stuart, D. *Acta Crystallogr.* 1983, **A39**, 158-166.
(17) *MolEN, An Interactive Structure Solution Procedure*; Enraf-Nonius: Delft, The Netherlands, 1990.
(18) Goodgame, D. M. L.; Khaled, A. M.; O'Mahoney, C. A.; Williams, D. J. *J. Chem. Soc., Chem. Commun.* 1990, 851.
(19) Goodgame, D. M. L.; Khaled, A. M.; O'Mahoney, C. A.; Williams, D. J. *Polyhedron* 1990, **14**, 1765.
(20) Kosturko, L. D.; Folzer, C.; Stewart, R. F. *Biochemistry* 1974, **13**, 3949.
(21) Palenik, G. J.; Koziol, A. E.; Katritzky, A. R.; Fan, W.-Q. *J. Chem. Soc., Chem. Commun.* 1990, 715-716.
(22) Palmer, M. H.; Blake, A. J.; Gould, R. O. *Chemical Physics* 1987, **115**, 219-227.
(23) Goldschmidt, G. H.; Llewellyn, F. J. *Acta Crystallogr.* 1950, **3**, 294-305.
(24) Soliman, E. M.; El-Roudi, A. M.; Hassaan, A. M. A.; Refaiy, S. A. *Synth. React. Inorg. Met.-Org. Chem.* 1989, **19**, 957-967.
(25) Goodgame, D. M. L.; Williams, D. J.; Winpenny, R. E. P. *Angew. Chem. Int. Ed. Engl.* 1987, **26**, 1044.
(26) Hansen, P. E. *Progress in NMR Spectroscopy* 1981, **14**, 175-296.
(27) Jovanovski, G.; Kamenar, B. *Acta Crystallogr. A* 1981, **37**, C171.
(28) Kamenar, B.; Jovanovski, G.; Grdenic, D. *Cryst. Struct. Commun.* 1982, **11**, 263-8.
(29) Marzilli, L. G.; Stewart, R. C.; Van Vuuren, C. P.; de Castro, B.; Caradonna, J. P. *J. Am. Chem. Soc.* 1978, **100**, 3967-8.
(30) Marzilli, L. G.; de Castro, B.; Caradonna, J. P.; Stewart, R. C.; van Vuuren, C. P. *J. Am. Chem. Soc.* 1980, **102**, 916-24.
(31) Marzilli, L. G.; de Castro, B.; Solorzano, C. *J. Am. Chem. Soc.* 1982, **104**, 461-6.

**Received: October 13, 1994 - Accepted: November 8, 1994 - Received in revised camera-ready format: November 25, 1994**

